# Targeted metagenomics unveils the molecular basis for adaptive evolution of enzymes to their environment

**DOI:** 10.3389/fmicb.2015.01018

**Published:** 2015-09-22

**Authors:** Hikaru Suenaga

**Affiliations:** Bioproduction Research Institute – National Institute of Advanced Industrial Science and Technology (AIST)Tsukuba, Japan

**Keywords:** targeted metagenomics, enzyme adaptation, environmental microbiology, directed evolution, high-throughput screening

## Abstract

Microorganisms have a wonderful ability to adapt rapidly to new or altered environmental conditions. Enzymes are the basis of metabolism in all living organisms and, therefore, enzyme adaptation plays a crucial role in the adaptation of microorganisms. Comparisons of homology and parallel beneficial mutations in an enzyme family provide valuable hints of how an enzyme adapted to an ecological system; consequently, a series of enzyme collections is required to investigate enzyme evolution. Targeted metagenomics is a promising tool for the construction of enzyme pools and for studying the adaptive evolution of enzymes. This perspective article presents a summary of targeted metagenomic approaches useful for this purpose.

## Introduction

Enzymes are the driving force behind life since they catalyze the biochemical reactions, and hence the metabolism, of all living organisms. Enzymes have evolved and been optimized for the metabolic networks of individual species (Copley, 2012). The pressure of survival at the metabolic level allows organisms to adapt to a changing chemical environment, such as the ability of bacteria to degrade xenobiotic compounds (Portnoy et al., 2011). There are many reports that microbes adapt to changes in their environment by improving their ability to degrade natural or xenobiotic compounds, and degradation enzymes play a crucial role in these adaptation mechanisms (Janssen et al., 2005). Therefore, in order to understand the ability of microorganisms to adapt rapidly to a new environment, it is necessary to understand how enzymes evolve to make this adaptation possible.

Comparison of the sequence and activity of enzymes from the same family but from different organisms indicates that enzymes are derived from a common ancestor and have accumulated mutations that allow them to adapt to environmental pressures. A collection or pool of related enzymes must be studied to understand enzyme evolution. There are two approaches for obtaining these specific enzyme pools: (i) construct the pool by directed evolution in the laboratory or (ii) retrieve the enzymes from the natural environment. Directed evolution, first used 20 years ago, mimics natural evolutionary processes (Stemmer, 1994; Dalby, 2011), allows the artificial evolution of enzymes in the laboratory under controlled selection pressures, and has resulted in the identification of different adaptive mechanisms (Arnold, 2001). Another approach is to isolate enzymes from microorganisms that show a specific enzymatic activity. For example, various homologous genes involved in the degradation of aromatic compounds have repeatedly been identified in microorganisms isolated from aromatics-contaminated environments (Furukawa et al., 2004; Vilchez-Vargas et al., 2010). These gene collections can also be useful for investigating molecular mechanisms in the adaptive evolution of xenobiotic-degrading enzymes and bacteria in the natural environment. However the majority of microorganisms in natural environments cannot be cultured using readily available technologies (Amann et al., 1995; Quince et al., 2008). This has spurred the development of metagenomics, which allows us to obtain various genes of interest from the entire microbial community (Handelsman, 2004; Shade et al., 2012). Metagenomics is, therefore, a powerful tool for constructing comprehensive gene collections of specific groups of enzymes from microbes in various habitats. This collection is useful for studying the adaptive evolution of enzymes and their host microorganisms.

## Two Strategies for Metagenomics

Metagenomics approaches are roughly classified into two groups: (i) whole metagenomics and (ii) targeted metagenomics, and are based on random and selective sequencing strategies, respectively. Many projects based on the random sequencing of microbial domains, such as the bacteria and archaea, and of viruses, have been reported (Thomas et al., 2012; Sharpton, 2014). Although whole metagenomic analyses revealed that microbial communities are well adapted to their geochemical conditions, those analyses provided no definitive evidence for the positive selection of enzymes for key ecological processes under environmental pressures. This lack of evidence is likely due to insufficient sequence data for the target enzyme group (Hemme et al., 2010). Mutations in the genes encoding such key enzymes would provide an adaptive phenotype optimized for a specific niche (Chattopadhyay et al., 2013). Therefore, high-resolution metagenomic sequencing to collect data of sufficient breadth and depth for any particular gene is necessary to verify the adaptive processes of enzymes in their ecosystem. This “targeted metagenomics” approach would be a suitable tool for constructing gene collections of specific groups of enzymes which are useful for studying their adaptive evolution. Previously, we presented a summary of the targeted metagenomics approaches to understanding the composition of gene clusters for key ecological processes in microbial communities (Suenaga, 2012). In this review, we focus on targeted metagenomics studies for surveying the adaptive evolution of enzymes toward environmental changes.

## Strategies for Targeted Metagenomics

In a targeted metagenomics approach, a deliberately selected DNA pool is sequenced. The selection process is usually based on (i) sequence-driven screening or (ii) function-driven screening. By focusing efforts on selective sequence analysis, targeted metagenomics can provide broad coverage and extensive redundancy of sequences for targeted genes and reveal specific genome areas directly linked to an ecological function, even at low abundances within a metagenome (Suenaga, 2012). Better sequence coverage of the obtained target metagenomics can be beneficial for genome assembly and subsequent data analysis. Examples of studies on targeted metagenomics are summarized below.

## Targeted Metagenomics Based on Sequence-driven Screening

The PCR-based approach has been used extensively to retrieve specific genes from a pool of DNA. Instead of cloning all the extracted DNA, primers are designed specifically against an identified target gene, such as phenol hydroxylase (Futamata et al., 2001), catechol 2,3-dioxygenase (Mesarch et al., 2000), and methane monooxygenase (Henckel et al., 2000). The advantage of using sequence-driven screening is that it uses well-established and high-throughput techniques, such as PCR and hybridization, and can be used for different targets. On the other hand, this approach requires designing DNA probes and primers derived from conserved regions of known gene or protein families. Thus, already-known sequence types will be identified and only a fragment of the main target gene will be amplified. Despite this limitation, combining PCR detection of small conserved regions with genome sequencing/walking at flanking regions makes it possible to obtain the entire gene and thus reconstruct the evolution of the target enzymes in response to alterations in the ecosystem.

Dissimilatory sulfate reduction is a crucial process in the mineralization of organic matter in marine sediments. PCR screening of a metagenomic fosmid library (11,000 clones) using degenerate primers resulted in the identification of three fosmid DNA fragments harboring a core set of essential genes for dissimilatory sulfate reduction; these fragments contained genes associated with the reduction of sulfur intermediates (*dsrAB* gene) and the synthesis of the prosthetic group of dissimilatory sulfate reductase (*aprA* gene; Mussmann et al., 2005). Complete sequence analysis of all fosmid inserts revealed the genomic context of the key enzymes of dissimilatory sulfate reduction as well as novel genes functionally involved in sulfate respiration in their flanking regions. The results support the hypothesis that the set of genes responsible for dissimilatory sulfate reduction was concomitantly transferred in a single event among prokaryotes.

Denitrification is a microbial respiratory process within the nitrogen cycle responsible for the return of fixed nitrogen to the atmosphere. A sequence-driven screening (colony hybridization) of 77,000 clones from a soil metagenomic library led to the identification of positive clones, and subsequent sequencing analysis revealed nine denitrification gene clusters (Ginolhac et al., 2004; Demanèche et al., 2009). This targeted metagenomics study indicated that the gene clusters involved in denitrification were probably subject to shuﬄing by endogenous gene displacement or by horizontal gene transfer between bacteria.

## Targeted Metagenomics Based on Function-driven Screening

Function-driven screening strategies potentially provide a means of revealing undiscovered genes or gene families that cannot be detected by sequence-driven approaches, although this screening is more laborious than sequence-based screening procedures (Ferrer et al., 2005; Fernández-Arrojo et al., 2010).

Nitrilases are important in synthesis and degradation for nitriles which are attractive starting compounds in the synthesis of fine chemicals. However, nitrilase genes are quite rare in bacterial genomes, and fewer than 20 were reported in the scientific and patent literature prior to the application of metagenomics (Podar et al., 2005). A leading metagenome company, Diversa Co. (USA), reported that 651 environmental samples collected worldwide from terrestrial and aquatic microenvironments were used to construct a metagenomics library, allowing identification of 137 new nitrilases by visual observation of *Escherichia coli* cells grown in liquid medium supplemented with nitrile substrate (Robertson et al., 2004). Phylogenetic analysis and enzymatic characterization of these enzymes revealed important correlations between sequence clades and selective properties of three structurally distinct nitrile substrates. Together with other metagenomic surveys for nitrilases (DeSantis et al., 2002; Bayer et al., 2011), the metagenomics approach has helped reveal the ecological distribution and diversity of nitrilases.

Deep-sea areas require that microbial communities adapt to harsh physical conditions, particularly high salinity and high pressure (Daffonchio et al., 2006; Smedile et al., 2013). A set of eight different enzymes was screened for activity from metagenomic fosmid and phage libraries constructed using DNA from five distinct deep-sea environments (Alcaide et al., 2015). The activities of the purified metagenomic proteins were characterized at various temperatures and salt conditions. The results suggested that adaptation to high pressure is linked to high thermal resistance in salt-saturated deep-sea conditions. Therefore, salinity might increase the temperature window for enzyme activity, and possibly microbial growth, in deep-sea habitats.

**FIGURE 1:**
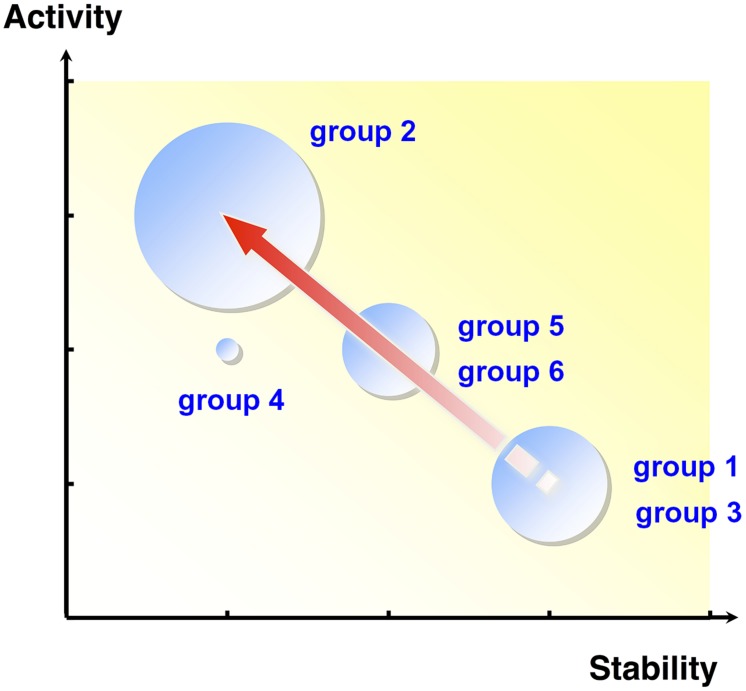
**The relationship between activity and thermostability of purified metagenomic extradiol dioxygenase (EDO) enzymes.** The size of each circle is proportional to the number of EDO enzymes in the group. The arrow indicates the proposed genetic evolutionary pathway. The thermostable ancestral groups, group 1 and 3, may have adaptively evolved toward the more active group 2 via group 5 and 6 by sacrificing unessential thermostability. EDO enzymes that acquired higher activities (group 2) were more frequently discovered in the retrieved enzyme collection.

Extradiol dioxygenases (EDOs) are enzymes that play an important role in the catabolism of aromatic compounds (Sipilä et al., 2008; Brennerova et al., 2009), cleaving the aromatic ring of catechol compounds, which are common intermediates in the aerobic microbial degradation of natural and xenobiotic aromatic compounds (Furukawa et al., 2004). Based on the activity of EDO enzymes, 96,000 fosmid clones were screened, and subsequent sequencing of positive fosmids led to the identification of 43 novel EDO genes (Suenaga et al., 2007, 2009). Using combinations of single nucleotide polymorphisms (SNPs), a possible evolutionary lineage of the EDO genes was constructed (**Figure 1**) and suggested that these genes evolved from a common ancestor (group 1 and 3), then diverged through the accumulation of various nucleotide mutations. Furthermore, investigation of the kinetic properties and thermal stability of the purified EDO enzymes showed an apparent trade-off between activity and stability (**Figure 1**). Bloom et al. (2006) reported that cytochrome P450 BM3 mutants with higher stabilities were more likely to acquire new or improved functions through random mutagenesis. They concluded that protein stability promotes adaptive protein evolution. Similarly, in EDO enzymes, the most thermostable ancestral groups (group 1 and 3) may have evolved toward more active groups (group 2 through group 5 and 6) by sacrificing thermostability. Note that EDO enzymes that had acquired higher activities (group 2 and 5) were more frequently discovered in the retrieved EDO clones, likely reflecting the allele frequencies in the environment.

The above studies of marine enzymes and EDO enzymes incorporated three-dimensional structural analyses to unveil the molecular mechanisms of enzyme adaptation, but the structural basis for enzyme evolution remains unclear. The amount of data on enzyme diversity made available by metagenomic approaches exceeds our ability to analyze the data based on our current knowledge of protein structure/function.

## Future Perspective

In the Section “Introduction”, I stated that directed evolution and metagenomics are different approaches for creating enzyme pools that can provide valuable hints on how enzymes adapt to ecological conditions. However, both approaches use the same key technology: high-throughput screening to collect the target enzymes. A variety of high-throughput screening methods have been established in recent years, and continue to develop in step with new developments in robotics, analytical devices, and visualizing assays. For example, microarray-based technologies coupled with microfluidic devices, cell compartmentalization, flow cytometry, and cell sorting have been proposed as promising new tools (Tracy et al., 2010; Simon and Daniel, 2011; Ekkers et al., 2012; Zhou et al., 2015). These screening systems offer higher levels of quantification and the possibility to detect multiple traits in one assay. Researchers in the two fields can share their wide knowledge of enzymes and up-to-date technologies to analyze enzyme characteristics.

Environmental pressures led to today’s diverse enzymes distributed throughout the earth’s ecosystems. Therefore, the collection of metagenomic enzyme pools from extreme environments, such as deep-sea hydrothermal vent fields, contaminated sites, and hot springs, is effective for studying the adaptive evolution of enzymes and their host microorganisms. In the near future, by integrating scientific knowledge in environmental microbiology, enzymology, and geology, it will be possible to assemble and use good quality enzyme collections suitable for the analysis of enzyme evolution.

## Conflict of Interest Statement

The author declares that the research was conducted in the absence of any commercial or financial relationships that could be construed as a potential conflict of interest.
